# Ketone Supplementation Decreased Lipopolysaccharide-Generated Anxiety-like Behavior in Female WAG/Rij Rats

**DOI:** 10.3390/ph19091359

**Published:** 2026-08-27

**Authors:** Enikő Rauch, Csilla Ari, Dominic P. D’Agostino, Zsolt Kovács

**Affiliations:** 1 Department of Biology, Berzsenyi Dániel Teacher Training Centre, ELTE Eötvös Loránd University, Károlyi Gáspár Tér 4., 9700 Szombathely, Hungary; 2 Institute of Biology, University of Pécs, Ifjúság Str. 6, 7624 Pécs, Hungary; 3 Ketone Technologies LLC, Tampa, FL 33612, USA; 4 Behavioral Neuroscience Research Laboratory, Department of Psychology, University of South Florida, Tampa, FL 33620, USA; 5 Laboratory of Metabolic Medicine, Department of Molecular Pharmacology and Physiology, Morsani College of Medicine, University of South Florida, Tampa, FL 33612, USA; 6 Institute for Human and Machine Cognition, Ocala, FL 34471, USA

**Keywords:** ketone supplement, LPS, anxiety, LDB test, female WAG/Rij rat

## Abstract

**Background/Objectives:** Inflammatory processes, including elevated pro-inflammatory cytokines such as interleukin 1β (IL-1β), have been implicated in the pathophysiology of anxiety disorders. The ketone body β-hydroxybutyrate (βHB) was shown to mitigate inflammatory processes induced by lipopolysaccharide (LPS) administration. It was also suggested that exogenous ketone supplements, such as ketone esters (KEs) and ketone salts (KSs), can decrease anxiogenic effects through increased blood ketone levels in the body (ketosis) in preclinical animal models. **Methods:** Thus, we investigated whether standard rodent food supplemented with KEKS (KEKS food: mix of a KE R/S-1,3-butanediol-acetoacetate diester and a KS Na^+^- and Ca^2+^- R/S-βHB salt in 1:1 ratio) for 7 days can alleviate the intraperitoneal (i.p.) 1 mg/kg LPS-generated anxiogenic effect in female Wistar Albino Glaxo/Rijswijk (WAG/Rij) rats. For the evaluation of anxiogenic effects, the light–dark box (LDB) test was used. **Results:** KEKS food significantly increased the blood R-β-hydroxybutyrate (R-βHB) level and decreased the blood glucose level compared to the baseline. Moreover, the LPS significantly (i) decreased the time spent in the light compartment, the number of chamber transitions, the number of re-entries to the light compartment and the number of rearing in the light compartment, and also (ii) increased the time of latency to the first re-entry to the light compartment compared to control. Nevertheless, KEKS food treatment abolished LPS-generated anxiety-like behavior. **Conclusions:** The current study demonstrated that a 7-day KEKS-supplemented diet can increase blood R-βHB levels and abolish acute LPS administration-evoked increases in anxiety levels in female WAG/Rij rats. Moreover, our results support the important role of neuroinflammation in pathological processes resulting in anxiety. Thus, these results suggest that exogenous ketone supplement-generated ketosis may be a promising therapeutic tool in the treatment of inflammatory process-mediated diseases, such as anxiety disorders.

## 1. Introduction

Anxiety disorders are highly prevalent, and their global burden has risen substantially [1]. Moreover, elevated anxiety is a common neuropsychiatric symptom of central nervous system disorders, such as epilepsy, depression, and Alzheimer’s disease [2,3]. However, our knowledge about the exact mechanism of action of this group of mental disorders is far from complete [4,5]. Pathological alterations have been demonstrated in GABAergic, glutamatergic, serotonergic, and noradrenergic neurotransmission within brain regions implicated in anxiety, including the hippocampus [6,7]. Different anxiolytic drugs, such as benzodiazepines, selective serotonin–norepinephrine reuptake inhibitors and selective serotonin reuptake inhibitors have variable efficacy and can generate several side effects [6,7,8]. In addition, many human patients with anxiety disorders showed pharmacological treatment resistance [9]. Consequently, further studies are needed to develop more effective and complementary therapeutic approaches for the treatment of anxiety disorders.

It has been demonstrated that inflammation is directly linked to anxiety [10]. For example, patients with anxiety disorders show elevated levels of pro-inflammatory cytokines, including tumor necrosis factor alpha (TNF-α) and interleukin 1β (IL-1β), relative to healthy controls [11,12,13]. The administration of several anxiolytic drugs and neuromodulation therapies can decrease neuroinflammation and the level of pro-inflammatory factors in connection with a reduction of anxiety symptoms [14,15]. Moreover, acute systemic lipopolysaccharide (LPS) administration in mice increased microglial activation and brain IL-1β and TNF-α levels, enhanced excitatory synaptic transmission in the basolateral amygdala, and elevated anxiety-like behavior [16]. These results confirm that inflammatory processes may have an essential role in pathological processes resulting in anxiety disorders through the dysregulation of synaptic transmissions and shifting of the excitatory/inhibitory balance to excessive excitation [16]. Consequently, different procedures alone or in combination with conventional anxiolytic drugs resulting in enhanced anti-inflammatory influences could be more effective for the treatment of patients with anxiety disorders compared to anxiolytic drugs alone.

Ketone bodies, particularly β-hydroxybutyrate (βHB), have been shown to attenuate inflammatory signaling, including reducing levels of pro-inflammatory cytokines such as IL-1β [17]. Moreover, βHB attenuated the LPS-generated increase in levels of IL-1β and TNF-α in microglial cells [18,19]. Exogenous ketone supplements, including ketone esters (KEs), ketone salts (KSs), medium-chain triglyceride (MCT) oil, and their combinations (KE + MCT, KE + KS, KS + MCT), have been shown to reduce anxiety-like behavior in preclinical models, in parallel with elevations in blood ketone levels (ketosis) [5,20,21,22,23,24,25,26,27]. These results suggest that ketosis generated by the administration of exogenous ketone supplements may be a promising and effective therapeutic tool in the treatment of human anxiety disorders [5,28], likely through anti-inflammatory influences. Indeed, it was confirmed that βHB administration can attenuate the increase in both the levels of pro-inflammatory cytokines and anxiety-like behavior [29,30].

Although anxiety disorders are approximately twice as prevalent in women as in men [4], the effects of exogenous ketone supplements on anxiety-like behavior have been studied predominantly in male animals [20,21,22,23,24,25,27]. Only a few studies have used female Wistar Albino Glaxo/Rijswijk (WAG/Rij) rats (e.g., Ref. 26) [26]. We previously reported that ketone supplement-evoked increases in blood R-β-hydroxybutyrate (R-βHB) levels differ between males and females, suggesting that ketosis-mediated effects on central nervous system disorders may be sex-dependent [31]. Thus, as a continuation of our previous study conducted on male rats [26,32], we investigated whether standard rodent food supplemented with KEKS (KEKS food: mix of a KE R/S-1,3-butanediol-acetoacetate diester and a KS Na^+^- and Ca^2+^- R/S-βHB salt in 1:1 ratio) for 7 days can alleviate the LPS-generated anxiogenic effect in female WAG/Rij rats. Putative changes in anxiety level were investigated using the light–dark box (LDB) test [33,34]. We hypothesized that LPS can enhance anxiety level and KEKS supplementation can abolish the LPS-generated changes in anxiety level (anxiety-like behavior) in female WAG/Rij rats.

## 2. Results

### 2.1. Influence of KEKS Food on Anxiety-like Behavior

**Figure 1:**
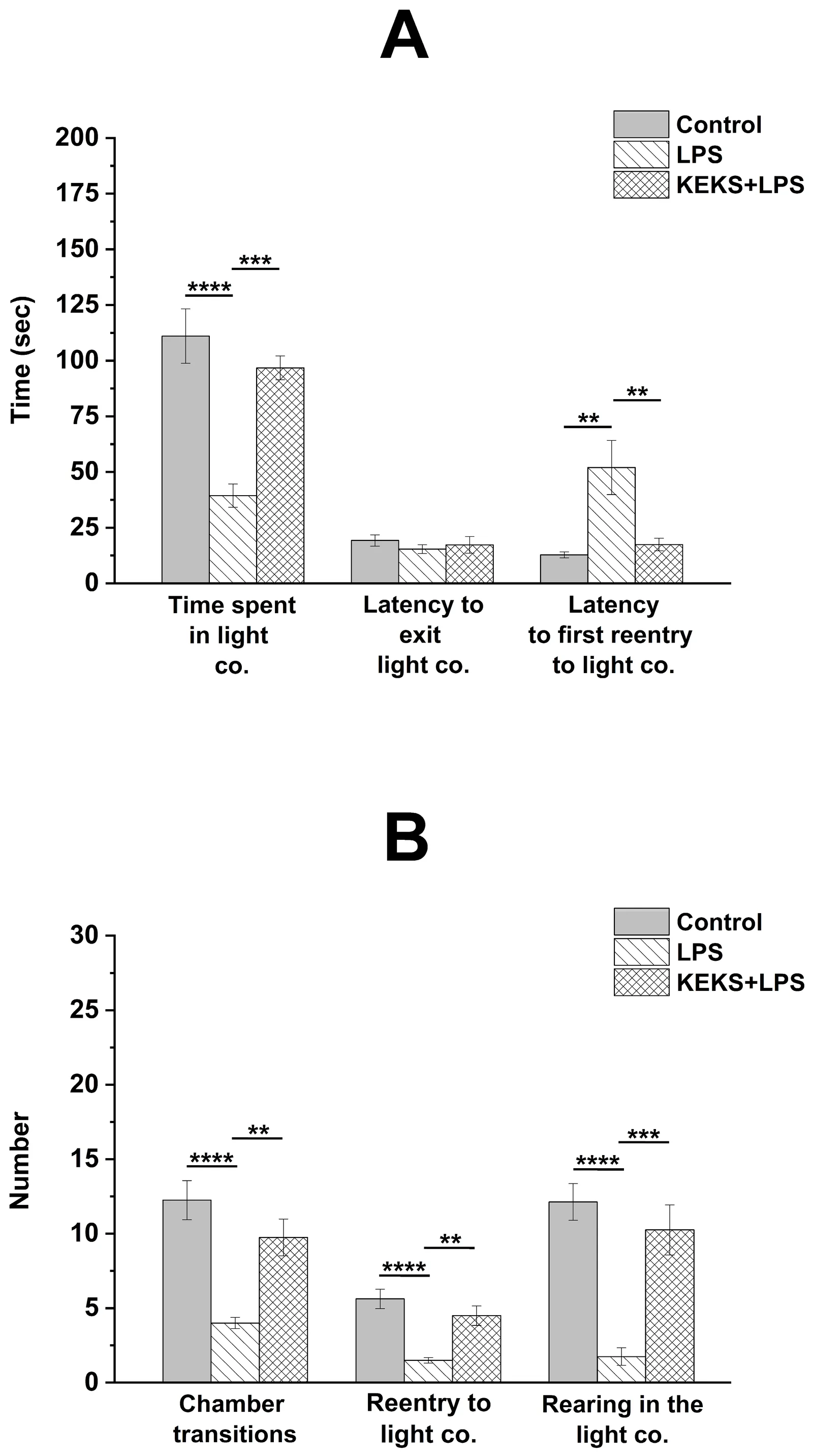
Effect of combined administration of standard rodent food and i.p. 1 mg/kg LPS (group 2/LPS group; *n* = 8) as well as KEKS-supplemented food and i.p. 1 mg/kg LPS (group 3/KEKS + LPS group; *n* = 8) for 7 days on anxiety level (LDB parameters) in female WAG/Rij rats compared to control (group 1/control; *n* = 8) (**A**,**B**). Abbreviations: co., compartment; KEKS, KEKS-supplemented food; LPS, lipopolysaccharide; ** *p* < 0.01; *** *p* < 0.001; **** *p* < 0.0001.

The intraperitoneal (i.p.) 1 mg/kg LPS significantly decreased the time spent in the light compartment, the number of chamber transitions, the number of re-entries to the light compartment and number of rearing in light compartment, compared to control (group 1/control group vs. group 2/LPS group; Figure 1A,B; Table 1). LPS treatment also significantly increased the time of latency to the first re-entry to the light compartment (group 1/control group vs. group 2/LPS group; Figure 1A; Table 1). Significant changes in time spent in the light compartment, time of latency to the first re-entry to the light compartment, the number of chamber transitions, the number of re-entries to the light compartment, and the number of rearing in the light compartment were not demonstrated after KEKS + LPS treatment compared to the control (group 1/control group vs. group 3/KEKS + LPS group; Figure 1A,B; Table 1). Nevertheless, KEKS + LPS treatment significantly decreased the LPS-generated influences on LDB test parameters (group 2/LPS group vs. group 3/KEKS + LPS group; Figure 1A,B). Consequently, KEKS food treatment for 7 days abolished all LPS treatment-evoked changes in the LDB test parameters and, therefore, the LPS-generated anxiogenic effect. However, the changes in time of latency to exiting the light compartment after the injection of LPS (group 2/LPS group) or the combined administration of KEKS food and LPS (group 3/KEKS + LPS group) were not significant compared to the control (group 1) (Figure 1A; Table 1).

### 2.2. KEKS Food Administration-Generated Effects on Blood Levels of R-βHB and Glucose, as Well as Body Weight

**Figure 2:**
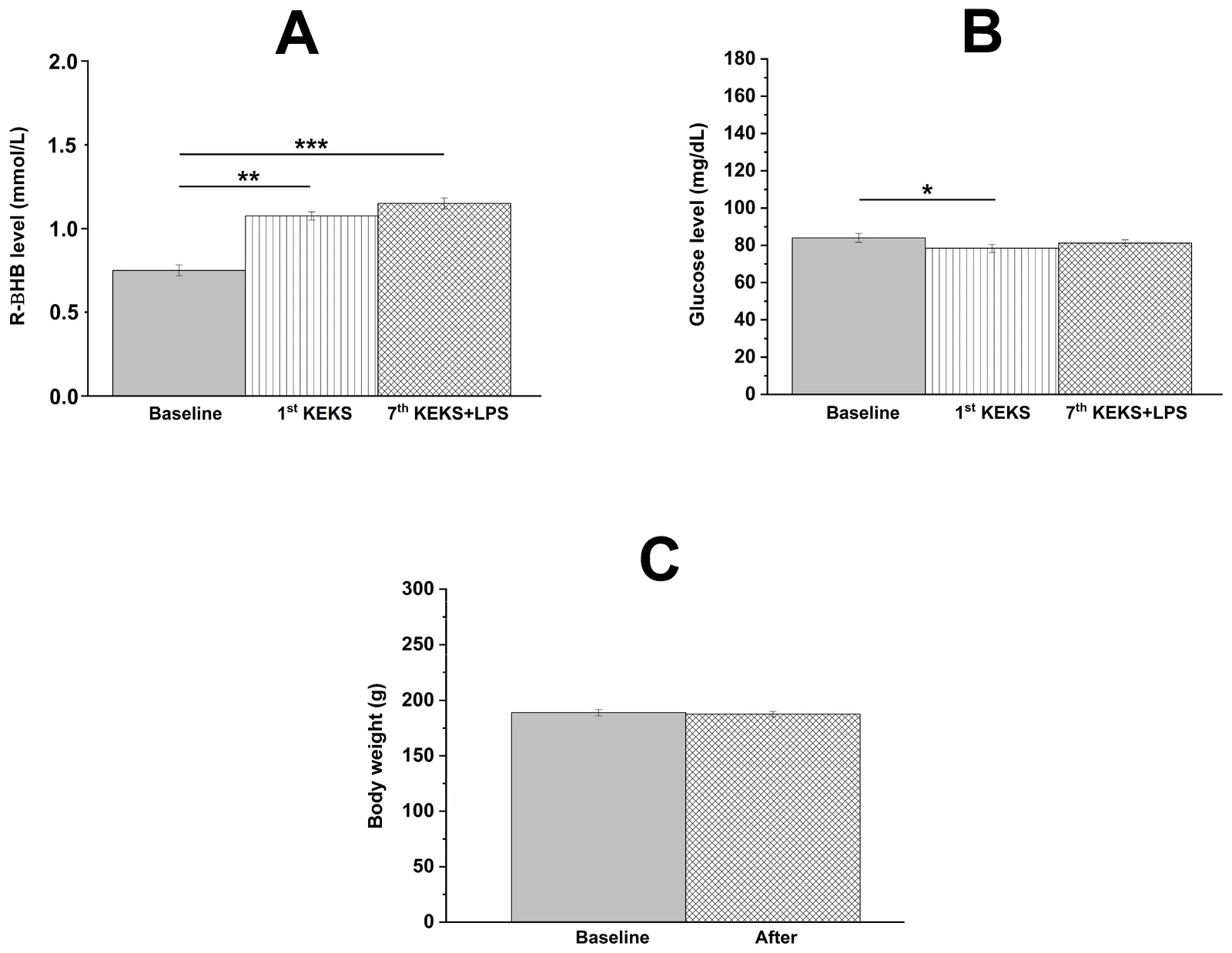
Effect of administration of KEKS-supplemented food and saline (on the first day: first KEKS) as well as KEKS-supplemented food and i.p. 1 mg/kg LPS (on the seventh day: seventh KEKS + LPS) on blood R-βHB (**A**) and glucose (**B**) levels in female WAG/Rij rats compared to baseline (group 3/KEKS + LPS group; *n* = 8). Influence of KEKS-supplemented food on body weight was also depicted (body weight was measured on the seventh day of KEKS food administration (after) and compared to baseline) (**C**). Abbreviations: KEKS, KEKS-supplemented food; LPS, lipopolysaccharide; R-βHB, R-beta-hydroxybutyrate; * *p* < 0.05; ** *p* < 0.01; *** *p* < 0.001.

It was demonstrated that both the first and seventh administrations of KEKS food (group 3/KEKS + LPS group) significantly increased the blood R-βHB level compared to the baseline (Figure 2A; Table 2). Nevertheless, this treatment was able to decrease the blood glucose level significantly only on the first administration day (Figure 2B; Table 2) compared to the baseline; however, when LPS was introduced, this effect was not present.

Moreover, similarly to our previous results [35,36], the body weight of rats did not change significantly after 7 days of administration of KEKS food compared to the baseline (*p* = 0.3506; Figure 2C). In this study, similarly to our previous works on the exogenous ketone supplement-evoked beneficial effects on WAG/Rij rats [35,36], we did not measure the food intake and the putative differences in KEKS food consumption among animals. Nevertheless, the body weight of animals treated with KEKS food (group 3/KEKS + LPS group) was unchanged (Figure 2C), suggesting that treatment by KEKS food did not exert its effect on LDB parameters and, therefore, on anxiety level through inadequate food intake and its consequences.

## 3. Discussion

In the present study, we demonstrated that KEKS food administration can increase the blood R-βHB level (Figure 2) and ameliorate LPS-induced changes in LDB test parameters (Figure 1). These preclinical results suggest that exogenous ketone supplementation exerts anxiolytic effects in female WAG/Rij rats, likely through the suppression of pro-inflammatory signaling.

A strong association between inflammatory processes and anxiety disorders has been suggested. For example, (i) an increased prevalence of anxiety disorders was demonstrated under inflammatory conditions, such as rheumatoid arthritis [37]; (ii) a positive correlation was revealed between pro-inflammatory cytokine (e.g., TNF-α) levels and the appearance of generalized anxiety disorder [38]; (iii) LPS-stimulated inflammation and increased baseline levels of peripheral inflammatory markers were positively correlated with the seriousness of the symptoms of anxiety [39,40]; (iv) the expression of pro-inflammatory markers was increased in genetically anxious mouse strains [41]; and (v) increased neuroinflammation was demonstrated in stress-induced anxiety [42]. Previous studies also showed that the peripheral injection of LPS—leading to the enhanced expression of pro-inflammatory cytokines such as IL-1β and TNF-α in the brain [43]—or pro-inflammatory cytokines can generate anxiety-like behavior, whose influence was demonstrated by different behavior tests, such as the LDB test [44,45,46]. Moreover, anti-inflammatory pretreatment was able not only to decrease neuroinflammation by attenuating the level of pro-inflammatory cytokines such as IL-1β and TNF-α but also prevent LPS-induced anxiety [47]. It was also demonstrated that the administration of IL-1β increased anxiety level in rats [48], whereas decreased anxiety-like behavior was recorded in interleukin 1 (IL-1) receptor null mutant mice [48].

The mechanisms by which inflammation induces anxiety remain incompletely understood. One proposal is that neuroinflammation perturbs neurocircuits, producing the dysfunctional activation and connectivity between brain regions implicated in anxiety, including the amygdala and hippocampus [49]. In relation to LPS-evoked anxiogenic influence, it has been demonstrated that LPS can augment the level of anxiety, likely through the activation of microglial cells, and therefore increase both pro-inflammatory cytokine and reactive oxygen species (ROS) release as well as enhanced excitability in implicated brain areas, such as the basolateral amygdala [16,49]. ROS and LPS have an important role in triggering the Toll-like receptor 4 (TLR4)/nucleotide-binding and oligomerization domain-like receptor pyrin domain-containing protein 3 (NLRP3) inflammasome signaling pathway. The multiprotein NLRP3 inflammasome contains NLRP3, the apoptosis-associated speck-like adaptor protein (ASC), and caspase-1 (a cysteine protease) [50]. The NLRP3 inflammasome can control caspase-1 activity and, therefore, the release of pro-inflammatory cytokine IL-1β. LPS triggers the activation of TLR4 and therefore evokes/enhances the NLRP3 inflammasome and pro-IL-1β expression through the nuclear factor kappa B (NF-kB)-dependent pathway. Finally, the activation of this signaling pathway can result in both the caspase-1-dependent cleavage of pro-IL-1β to active IL-1β as well as the enhanced release of IL-1β [50,51]. As was demonstrated, this kind of enhancement of neuroinflammatory processes-generated changes can lead to increased anxiety level [44,52]. Moreover, it was also revealed that i.p.-injected LPS can generate an increase in microglial activity and NLRP3 expression and, therefore, the release of pro-inflammatory cytokines (e.g., IL-1β and TNF-α) in the hippocampus in mice [53]. These results suggest the important role of the microglial TLR4/NF-kB/NLRP3 inflammasome/IL-1β pathway in LPS-generated anxiety. Indeed, it was confirmed that the inhibition of both the NLRP3 inflammasome [54,55] and TLR4 [56] attenuates anxiety-like behavior, whereas the activation of the NLRP3 inflammasome was demonstrated in response to anxiety [57] in mice. Moreover, LPS-induced increases in ROS have been proposed to elevate pro-inflammatory cytokine levels [58] and, in turn, anxiety-like behavior.

Ketone bodies such as βHB have been shown to be much more than metabolites serving as an alternative energy source for the brain tissue: these molecules play an important role in cellular signaling processes as well [59]. For example, βHB acts as an endogenous NLRP3 inhibitor, reducing the expression of NLRP3, ASC, caspase-1, and IL-1β and suppressing IL-1β release in both human monocytes and mouse models [17]. The administration of βHB suppressed the spinal cord injury-induced activation of the NLRP3 inflammasome and decreased the expression of IL-1β [60]. Furthermore, βHB reduced the LPS-generated increase in IL-1β and TNF-α mRNA expression, as well as IL-1β and TNF-α levels in microglial cells by inhibiting NF-kB signaling through G-protein-coupled receptor 109A (GPR109A) [18,19]. It was also demonstrated in LPS-treated mice that βHB can downregulate TNF-α expression in the brain [61]. Exogenous KE administration, prior to LPS-injection, generated ketosis and produced protective effects against the development of systemic inflammation (e.g., decreased the level of pro-inflammatory cytokines) in mice [62]. Consequently, it is possible that the administration of exogenous ketone supplements, such as KEKS food, can evoke not only ketosis but also a decrease in LPS-generated inflammatory processes resulting in anxiolytic influence through modulation (inhibition) of the TLR4/NF-kB/NLRP3 inflammasome/IL-1β signaling pathway. Indeed, this hypothesis was confirmed by our results demonstrating that KEKS food increased blood R-βHB level (Figure 2) and abolished the LPS-generated increase in anxiety-like behavior (Figure 1) in female WAG/Rij rats.

It has also been demonstrated that ketosis/βHB may diminish ROS production [61] while increasing brain adenosine levels [63]. ROS can increase the release of pro-inflammatory cytokines [58]. Adenosine can attenuate not only LPS-induced cytokine production through A2A type of adenosine receptors (A2ARs) in microglial cells [64] but also LPS-generated increase in the expression of other genes involved in inflammation, such as inducible nitric oxide synthase (iNOS). Adenosine exerts its last influences likely through A1 and A3 types of adenosine receptors (A1Rs and A3Rs) in astrocytes [65]. These results suggest that KEKS-induced changes in ROS and adenosine levels may also attenuate LPS-induced, inflammation-mediated increases in anxiety-like behavior.

In this study, it has been demonstrated that LPS treatment generated a significant (i) decrease in the time spent in the light compartment, the number of chamber transitions, the number of re-entries to the light compartment, and the number of rearing in the light compartment; meanwhile, (ii) there was an increase in latency to the first re-entry into the light compartment compared to control in female WAG/Rij rats (Figure 1). These influences of LPS injections were abolished by pretreatment of KEKS food administration (Figure 1). Based on the results above and studies using the LDB test [66,67], as well as WAG/Rij rats [20,25,26] for investigation of treatment-generated changes in anxiety level, we can assume the anxiolytic effect of exogenous ketone supplements against LPS-generated anxiogenic influence. Moreover, it was also suggested that the increased time spent in the light compartment is the most reliable sign of decreased anxiety [33,68,69]. Supplementation of KEKS food attenuated the LPS-generated decrease in time spent in the light compartment (Figure 1A), confirming the anxiolytic influence of exogenous ketone supplements. Nevertheless, after LPS administration, the demonstration of the significant decrease in the number of chamber transitions and the number of rearing in the light compartment (Figure 1B) may refer to a decrease in both locomotor activity and the exploratory behavior of rats [34,67]. Previously, similarly to our recent results, a parallel increase in anxiety level and decrease in locomotor activity were demonstrated in WAG/Rij rats [70], suggesting a possible close connection between treatment-generated changes in anxiety level and locomotor activity. However, all these effects were also ameliorated by the administration of KEKS food (Figure 1B), suggesting the beneficial effect of ketone supplementation on both locomotor activity and exploratory behavior. Indeed, for example, it was demonstrated that the intragastric gavage of exogenous ketone supplements, such as KE, KS, and KEKS induced ketosis and, therefore improved impaired motor performance and exploratory behavior in mice and/or rat preclinical models, such as WAG/Rij rats [71,72]. It has been demonstrated that LPS administration can generate impaired (reduced) locomotor activity and exploratory behavior [48,67,73,74,75], likely through increased levels of pro-inflammatory cytokines, such as IL-1β [48,75,76,77]. Consequently, it might be speculated that the administration of KEKS food had a beneficial influence on LPS-generated harmful effects, such as (i) impairment in locomotor functions as well as explorative behavior and (ii) increased anxiety level, likely through the inhibition of inflammatory processes.

There are several limitations of our study. We used only 8-month-old female WAG/Rij rats and one method (the LDB test) for investigation of the effect of KEKS food on LPS-induced changes in anxiety level (anxiety-like behavior). Moreover, changes in ovarian cycle and hormone levels, as potential influencing factors on behavior [78], as well as inflammatory markers and cells were not investigated. Nevertheless, an important goal was to extend our previous studies on the influence of ketone supplement administration-generated changes in the level of anxiety using similarly aged female WAG/Rij rats and using a similar method [26]. Moreover, the LDB test is a well-known and popular method in pharmacology for investigating the effect of different drugs on anxiety level in rats, often used as a single method [33,66,69,79,80]. However, to evaluate and extend our results in relation to ketone supplements-generated beneficial effects on LPS-induced alterations in anxiety level, further studies will be needed. For example, (i) we can also use other methods (e.g., elevated plus maze test), different age groups of rats, and different model animals (e.g., mice); (ii) we can measure, for example, the level of other ketone bodies (e.g., acetoacetate) in addition to βHB, as well as the level of ovarian/sex hormones and inflammatory markers; (iii) we can investigate the effect of different doses of other ketone supplements and their mixes (e.g., KS, MCT, and KSMCT) on anxiety level to identify the most effective formulations and doses; and (iv) we can reveal the exact mechanisms of action of ketone supplementation-evoked ketosis on LPS-generated changes in anxiety level by, amongst others, the investigation of treatment-generated changes on different neurotransmitter systems, such as serotonergic and dopaminergic systems [67].

## 4. Materials and Methods

### 4.1. Animals

Eight-month-old female WAG/Rij rats (*n* = 24; 184–201 g; from the breeding colony at Eötvös Loránd University, BDTTC, Szombathely, Hungary) were housed in groups (four animals in each group). Standard animal housing conditions (free access to standard rodent food SSNIFF RM-Z + H rat diet (TOXI-COOP Ltd., Budapest, Hungary) and water; air-conditioned room (22 ± 2 °C); 12:12 h light–dark cycle: between 08.00 AM and 08.00 PM light was on) were provided for rats. After the last treatment, the animals were euthanized in isoflurane (5% isoflurane; vaporizer: Stoelting Co., Wood Dale, IL, USA).

### 4.2. Light–Dark Box Test

The LDB test is an often-used method to investigate the anxiolytic influence of treatments in rodent models [33,34]. The LDB test is based on a conflict between the propensity of the rodents to explore the environment and to avoid brightly lit places [68,69]. It has been suggested previously that an increase in time spent in the light compartment, latency to exit the light compartment, number of chamber transitions, and number of rearing in the light compartment as well as a decrease in latency to first re-entry into the light compartment are associated with a treatment-generated reduction in anxiety level (anxiety-like behavior) [33,34,66,68,69].

The Plexiglas LDB apparatus was separated into light and dark chambers (boxes/compartments). The light and uncovered chamber (light box/compartment) was transparent, whereas the dark, black chamber was covered with a black cover (dark box/compartment). Between the two compartments, a rectangular opening was created. Illumination of the light box was 80 lux, whereas the dark box illumination was under 5 lux (practically not observable) [26]. LDB tests were carried out between 4.00 P.M. and 6.00 P.M.

At the start of the LDB test, the animal was placed at the center of the light compartment facing away from the opening of the apparatus and a 5 min of free exploration (behavior) was video recorded in the light compartment. After all sessions, the apparatus was cleaned with 30% ethanol solution and water. Video recordings were used to measure (i) the time spent in the light compartment; (ii) the latency to first exit the light compartment; (iii) the latency to first re-entry into the light compartment; (iv) the number of chamber transitions (if all four paws of the rat were in the opposite chamber, the entry was considered; all chamber transitions from light to dark compartment and vice versa, the number of re-entries to the light compartment was depicted separately); and (v) the number of rearing in the light compartment [26,34,69].

### 4.3. Treatments

Acute LPS administration in animals (e.g., mice and rats) is a widely accepted method to investigate the relationship between neuroinflammation-generated processes and anxiety [52]. Indeed, for example, i.p. administration of 1 mg/kg LPS generated an increase in anxiety level [73]. Moreover, it was also demonstrated previously that a ketone supplementation-evoked increase in R-βHB level (ketosis) can generate an anxiolytic effect without side effects [20,25,26] in WAG/Rij rats. To investigate the influence of ketone supplementation on LPS-generated changes in anxiety level (anxiety-like behavior), we used i.p. 1 mg/kg LPS (*E. coli*, serotype O111:B4; Sigma-Aldrich, Inc., Budapest, Hungary), and rats were fed with fresh paste-like KEKS food ad libitum. The ingredients of KEKS food mix were (i) powdered standard rodent food, (ii) 10% KE (R,S-1,3-butanediol—acetoacetate diester; % by weight) and 10% KS (Na^+^- and Ca^2+^- R/S-βHB salt; % by weight), (iii) 1% saccharin (Thermo Fisher Scientific, Waltham, MA, USA) to increase palatability, and (iv) water [81]. KE and KS were synthesized at the University of South Florida (USF, Tampa, FL, USA) in collaboration with SavInd, Inc. (Urbana, IL, USA) [24,82]. We provided free access to fresh KEKS-supplemented food in a Petri dish every day placed at the bottom of each animal’s cage [81]. It was demonstrated that ketone supplementation alone significantly increased the level of R-βHB and decreased the anxiety level in WAG/Rij rats [20,25,81]. Thus, treatment by KEKS food alone and the investigation of its influence on anxiety level were not repeated in this study.

**Figure 3:**
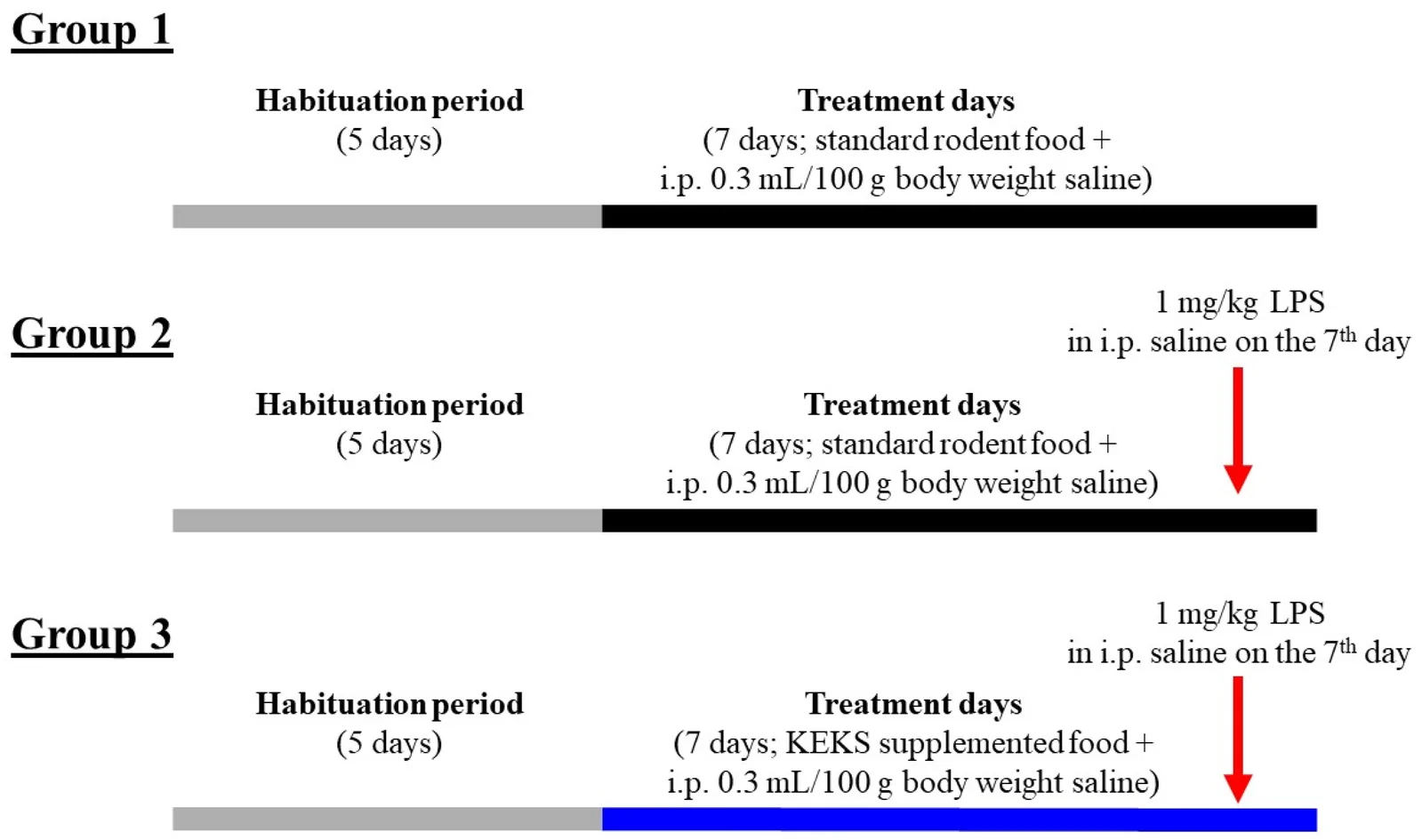
Treatments of WAG/Rij rats (*n* = 24; eight animals in each group). KEKS-supplemented food contained powdered standard rodent chow,10% KE (R,S-1,3-butanediol—acetoacetate diester; % by weight), 10% KS (Na^+^- and Ca^2+^- R/S-βHB salt), 1% saccharin and water. Abbreviations: i.p., intraperitoneal; KEKS, KEKS-supplemented food; LPS, lipopolysaccharide. Black line: standard rodent food during treatment days; Blue line: KEKS supplemented food during treatment days.

After the habituation period (handling the animals in the treatment room once daily for 5 days), rats were assigned to three groups based on the random allocation of animals to the groups by a computer-based random order generator. Animals of group 1 (*n* = 8) were fed by paste-like standard rodent food (without ketone supplements) and injected i.p. by 0.3 mL/100 g body weight saline on 7 consecutive days (group 1/control group) (Figure 3). The second group (*n* = 8) received similar treatments to animals of the first group but the last (7th) i.p injection contained 1 mg/kg LPS in 0.3 mL/100 g body weight saline (group 2/LPS group) (Figure 3). The third group of rats (*n* = 8) received paste-like KEKS-supplemented food for 7 days. Animals in the third group received i.p. saline injection (0.3 mL/100 g body weight) for 6 days. On the seventh day, these animals were injected with i.p. 1 mg/kg LPS in 0.3 mL/100 g body weight saline (group 3/KEKS + LPS group) (Figure 3). In all groups, LDB tests were performed for 5 min on the seventh day of the treatment (180 min after the last i.p. injection of saline (group 1) or LPS (group 2 and group 3)) [73].

The influence of KEKS food on blood glucose and R-βHB levels (group 3) was determined on the last adaptation day (baseline level), as well as the first and last KEKS food treatment days (in the latter case, about 5 min after the LDB test) using a blood glucose and ketone monitoring system (Precision Xtra™, Abbott Laboratories, Alameda, CA, USA) [20,26]. For the blood test, blood was taken from the tail vein of animals.

The body weight of the rats was also measured two times: on the last adaptation day (baseline) and the last KEKS food treatment day (group 3).

### 4.4. Statistics

All data were presented as mean ± standard error of the mean (S.E.M.). The treatment-generated changes in blood R-βHB and glucose levels, LDB parameters, as well as body weight were compared to baseline or control levels. Version 9.2.0 of the GraphPad Prism software, as well as a two-way ANOVA (Analysis of Variance) with Tukey’s multiple comparisons test and *t*-test, were applied for data analysis. The results were considered significant when *p* < 0.05. Statistical evaluation of all collected data was carried out in a blinded fashion (the observers were blinded to the treatment allocation while analyzing behavioral data). After all data were analyzed, deblinding was performed.

## 5. Conclusions

The present study confirmed and extended our previous findings in female WAG/Rij rats. A 7-day KEKS-supplemented diet significantly increased blood R-βHB levels and produced anxiolytic effects in the light–dark box test, both under baseline conditions and following acute LPS administration. These results support a role for neuroinflammation in the pathogenesis of anxiety and suggest that supplement-induced nutritional ketosis may have therapeutic potential in central nervous system disorders in which neuroinflammation contributes to symptom expression. Moreover, these findings suggest that ketogenic metabolic therapy (KMT) combined with standard food may represent a translationally relevant, metabolism-based approach to anxiety disorders. This conclusion carries an important qualification: the formulation tested here contains a 1,3-butanediol-containing ketone ester, and recent work reports adverse hepatic effects with the chronic administration of 1,3-butanediol-containing supplements [83,84]. Because our study used a 7-day protocol, it does not address chronic safety. Future work should therefore evaluate whether comparable anxiolytic effects can be achieved with formulations that exclude 1,3-butanediol, and should also establish optimal dose, duration, and safety margins before clinical translation. However, several questions were unanswered. The mechanisms underlying the protective effect of ketone supplementation against LPS-induced changes were not directly assessed here. Interpreting these findings will require the measurement of inflammatory cytokines and inflammasome markers, microglial activity, and neurotransmitter system function alongside anxiety-like behavior across LPS, ketone supplementation, and combined administration. Whether these effects generalize also remains open: future work should test other ketone supplements, particularly ketone salts, using additional behavioral paradigms, such as the elevated plus maze test and additional rodent models.

## Figures and Tables

**Table 1:** Effect of the combined administration of standard rodent food and i.p. 1 mg/kg LPS (group 2/LPS group) as well as KEKS-supplemented food and i.p. 1 mg/kg LPS (group 3/KEKS + LPS group) for 7 days on anxiety level in female WAG/Rij rats compared to control (standard rodent food + saline, group 1). KEKS-supplemented food contained powdered standard rodent chow, 10% KE (R,S-1,3-butanediol—acetoacetate diester; % by weight), 10% KS (Na^+^- and Ca^2+^- βHB salt), 1% saccharin, and water. Abbreviations: LPS, lipopolysaccharide; ns, non-significant; ** *p* < 0.01; **** *p* < 0.0001.

	Standard Rodent Food + Saline (Group 1/Control Group; Mean ± S.E.M.)	Standard Rodent Food + LPS (Group 2/LPS Group; Mean ± S.E.M.; Level of Significance/*p*-Value)	KEKS-Supplemented Food + LPS (Group 3/KEKS + LPS Group; Mean ± S.E.M.; Level of Significance/*p*-Value)
Time spent in light compartment (sec)	111.0 ± 12.17 -	39.4 ± 5.28 ****/<0.0001	96.8 ± 5.30 ns/0.454
Latency to exit light compartment (sec)	19.3 ± 2.57 -	15.4 ± 1.98 ns/0.6116	17.3 ± 3.76 ns/0.8751
Latency to first re-entry to light compartment (sec)	12.8 ± 1.3195 -	52.0 ± 12.17 **/0.0027	17.4 ± 2.78 ns/0.8944
Number of chamber transitions	12.3 ± 1.31 -	4.0 ± 0.38 ****/<0.0001	9.8 ± 1.24 ns/0.2411
Number of re-entries to light compartment	5.6 ± 0.65 -	1.5 ± 0.19 ****/<0.0001	4.5 ± 0.66 ns/0.3298
Number of rearing in the light compartment	12.1 ± 1.23 -	1.8 ± 0.59 ****/<0.0001	10.3 ± 1.69 ns/0.5498

**Table 2:** Influence of combined administration of KEKS-supplemented food and saline (on the first day) as well as combined administration of KEKS-supplemented food and i.p. 1 mg/kg LPS (on the seventh day) on blood R-βHB and glucose levels in female WAG/Rij rats compared to baseline (group 3/KEKS + LPS group). KEKS-supplemented food contained powdered standard rodent chow, 10% KE (R,S-1,3-butanediol—acetoacetate diester; % by weight), 10% KS (Na^+^- and Ca^2+^- βHB salt), 1% saccharin and water. Abbreviations: KEKS, KEKS-supplemented food; LPS, lipopolysaccharide; ns, non-significant; R-βHB, R-beta-hydroxybutyrate; * *p* < 0.05; ** *p* < 0.01; *** *p* < 0.001.

	Blood R-βHB Level (Group 3/KEKS + LPS Group); mmol/L; Mean ± S.E.M.; Level of Significance/*p*-Value)	Blood Glucose Level (Group 3/KEKS + LPS Group; mg/dL; Mean ± S.E.M.; Level of Significance/*p*-Value)
Baseline	0.75 ± 0.03 -	84.00 ± 2.38 -
First KEKS-supplemented food + saline	1.08 ± 0.03 **/<0.0011	78.38 ± 2.20 */0.0228
Seventh KEKS-supplemented food + LPS	1.15 ± 0.03 ***/0.0005	81.25 ± 1.69 ns/0.3145

## Data Availability

The original contributions presented in this study are included in the article. Further inquiries can be directed to the corresponding author.

## Abbreviations

ASC	apoptosis-associated speck-like protein
βHB	β-hydroxybutyrate
IL-1β	interleukin 1β
i.p.	intraperitoneal
KE	ketone ester
KEKS	a mix of KE and KS
KEMCT	a mix of KE and MCT oil
KMT	ketogenic metabolic therapy
KS	ketone salt
KSMCT	a mix of KS and MCT oil
LDB	Light–dark box
LPS	lipopolysaccharide
MCT	medium chain triglyceride
NF-kB	nuclear factor kappa B
NLRP3	nucleotide-binding and oligomerization domain-like receptor pyrin domain-containing protein 3
R-βHB	R-β-hydroxybutyrate
ROS	reactive oxygen species
TLR4	Toll-like receptor 4
TNF-α	tumor necrosis factor alpha
WAG/Rij	Wistar Albino Glaxo/Rijswijk
